# How psychosocial and economic impacts of COVID-19 pandemic can interfere on bruxism and temporomandibular disorders?

**DOI:** 10.1590/1678-7757-2020-0263

**Published:** 2020-05-11

**Authors:** Camila Megale ALMEIDA-LEITE, Juliana STUGINSKI-BARBOSA, Paulo César Rodrigues CONTI

**Affiliations:** 1 Universidade Federal de Minas Gerais Instituto de Ciências Biológicas Departamento de Morfologia Belo HorizonteMG Brasil Universidade Federal de Minas Gerais, Instituto de Ciências Biológicas, Departamento de Morfologia, Belo Horizonte, MG, Brasil.; 2 Universidade de São Paulo Bauru Orofacial Pain Group BauruSP Brasil Universidade de São Paulo, Bauru Orofacial Pain Group, Bauru, SP, Brasil.; 3 Universidade de São Paulo Faculdade de Odontologia de Bauru Departamento de Prótese e Periodontia BauruSP Brasil Universidade de São Paulo, Faculdade de Odontologia de Bauru, Departamento de Prótese e Periodontia, Bauru, SP, Brasil.

In January 2020, a novel coronavirus has been announced as the etiologic pathogen of COVID-19 disease that had become a major epidemic threat in China. It has widely spread since December 2019 not only in China but also in many countries around the world, which became a major challenge for public health.^1 , 2^ World Health Organization (WHO) announced COVID-19 outbreak a pandemic in March 2020 and it constitutes a public health emergency of international concern.^3^ As of April 20, 2020, there have been more than 2.3 million confirmed cases and 157.000 deaths globally.^4^ COVID-19 consequences on the global economy and financial crisis are already tangible. Quarantines, disruptions of daily life, travel, work, school education and social isolation that occurred worldwide may have impacting consequences on mental health.^5^

Previous public health emergencies have demonstrated to have an influence on mental health.^6^ Literature shows that psychological reactions to previous epidemics and pandemics depend on individual vulnerability such as intolerance of uncertainty, perceived vulnerability to disease, and anxiety.^6^ In the current situation, there are many uncertainties concerning SARS-CoV-2 origin, nature, government capacity to prevent the spread of infection, and seriousness of the risk.^7^ Moreover, the lack of faith in the healthcare system to deal with new cases, worries about becoming infected, fear of death, increase in hygienic and avoidance behaviors, lack of information and misinformation fuel excessive fear and create an environment of anxiety and depression that interfere with basic daily activities, including sleep quality.^7 , 8 , 9^ In addition, people who are quarantined lose social connections and feelings of loneliness and anger may develop.^10 , 11^ It has already been well documented the strong impact that COVID-19 is having on the psychological issues in China, where a significant portion of population has reported moderate-to-severe anxiety.^12^ Medical health-care workers, mainly females, are also facing increased levels of anxiety and stress.^9 , 13^

It is well established the importance of psychosocial factors in development and maintenance of Temporomandibular Disorders (TMD) and the high prevalence of psychological disturbances in TMD patients, mainly in those who suffer from masticatory muscle disorders.^14 - 16^ Moreover, there is a significant relationship between painful TMD, depression, and anxiety.^16 - 19^ All psychological issues involved in emergency and threatening situations like the ones faced with COVID-19 pandemic are able to trigger a chain of events that culminate with higher levels of sympathetic activity and further release of adrenocortical steroids which lead to muscle vasoconstriction and increased peripheral vascular resistance. Feelings of warmth and cold, palpitations, tachycardia, nausea, abdominal pain, diarrhea, and constipation can all be the consequences of autonomic stress responses.^20^ All these events are supposed to create/perpetuate a situation of system overloading, a common finding in TMD patients. The autonomic impairment may also lead to increased sympathetic drive and sensation of hyperarousal which create and perpetuate any sleep disturbance.^21^ If maintained, this cycle may play an important role in pain maintenance, especially in psychological vulnerable individuals. Hence, the occurrence of post-pandemic signs and symptoms of chronic orofacial pains, including TMD, is expected in a very similar pattern to well described posttraumatic stress syndrome.

The association between bruxism and psychological aspects has been documented,^22 - 27^ although the intensity of sleep bruxism has not been associated to self-reported stress, depression, TMD or TMD-related pain.^22 - 24^ A recent systematic review, however, reported that some specific symptoms of the anxiety disorders spectrum might have association to probable sleep bruxism.^25^ Awake bruxism, in contrast, has psychosocial factors such as anxiety, stress and difficulty in identifying and describing feelings as important as somatic causes in its occurrence and maintenance.^26^ Patients with high levels of stress are almost 6 times more likely to report awake bruxism.^27^ Sustained muscle contraction of head and neck is also related to a required body posture associated to fight-or-flight response. Therefore, muscle contraction in awake bruxism could be part of the defense behavior associated with anxiety and stress.^28^ The anxiety-related processes occur in the CNS and involves interactions among prefrontal cortex, limbic, paralimbic structures (amygdala, insula, anterior cingulate gyrus) and motor regions of the brain stem that leads to motor and physiological responses not only to stress, but also increased alertness and attention.^29^ Under non-stress conditions, the prefrontal cortex (PFC) regions regulate behavior, thought and emotion, including inhibition of inappropriate motor responses. However, in stressful conditions, the amygdala activates pathways in the hypothalamus and brainstem and impairs PFC regulation.^30^

Moreover, it is important to highlight that some other long-term effects of COVID-19 may be described in the future and deserve attention. Viral infections in the nervous system may lead to meningoencephalitis and neuropathies as seen for herpesviruses, Zika virus, and human immunodeficiency virus (HIV).^31 , 32 , 33^ Since SARS-CoV-2 infection has caused central nervous system manifestations,^34 , 35^ possible consequences as neuropathic pain states may also be a possible long term manifestation of the pandemic.

In conclusion, COVID-19 outbreak may lead to major impacts in applied oral sciences for the next years. Remarkably, it could be expected that psychological factors associated to pandemic may lead to a greater risk of developing, worsening and perpetuating bruxism (mainly awake bruxism) and TMD. Orofacial pain specialists should be aware of this fact. Guidelines for patient’s education, self-management, home care and relaxations techniques are already available on the WEB and are useful tools in times of social isolation and pain.
